# Supplementary material for: Mechanisms governing the pioneering and redistribution capabilities of the non-classical pioneer PU.1
Source: Nat Commun. 2020 Jan 21;11:402. doi: 10.1038/s41467-019-13960-2 (PMC6972792; doi:10.1038/s41467-019-13960-2)
Supplement: Supplementary file 7 — Source data [file 41467_2019_13960_MOESM7_ESM.zip › Source_Data/FigureS5/FigureS5A_MotifsCommonPeaks/homerResults/motif42.similar.html]

motif42

## Information for motif42

C
A
G
T
A
T
G
C
T
G
C
A
A
G
T
C
C
T
A
G
A
C
G
T
T
A
C
G
T
C
G
A
G
A
T
C
A
G
C
T
  
Reverse Opposite:  

T
C
G
A
C
T
A
G
A
G
C
T
A
T
G
C
C
G
T
A
A
G
T
C
C
T
A
G
A
C
G
T
T
A
C
G
G
T
C
A
  

|  |  |
| --- | --- |
| p-value: | 1e-28 |
| log p-value: | -6.485e+01 |
| Information Content per bp: | 1.629 |
| Number of Target Sequences with motif | 539.0 |
| Percentage of Target Sequences with motif | 6.07% |
| Number of Background Sequences with motif | 1287.0 |
| Percentage of Background Sequences with motif | 3.39% |
| Average Position of motif in Targets | 99.7 +/- 57.3bp |
| Average Position of motif in Background | 99.1 +/- 62.5bp |
| Strand Bias (log2 ratio + to - strand density) | 0.1 |
| Multiplicity (# of sites on avg that occur together) | 1.05 |
| Motif File: | file (matrix) reverse opposite |

### Similar de novo motifs found

|  |  |  |  |  |  |  |  |
| --- | --- | --- | --- | --- | --- | --- | --- |
| Rank | Match Score | Redundant Motif | P-value | log P-value | % of Targets | % of Background | Motif file |
| 1 | 0.865 | C T A G G A C T A G T C G C T A A T G C C T A G A G C T C T A G T C G A A T G C T C A G | 1e-17 | -39.978561 | 1.46% | 0.53% | motif file (matrix) |
| 2 | 0.866 | C T G A A G T C A C T G A G C T C T A G C T G A G A T C | 1e-16 | -39.141365 | 9.62% | 6.91% | motif file (matrix) |
| 3 | 0.761 | C G A T C A G T T C G A A G T C A C T G A C G T T G C A | 1e-14 | -32.862010 | 2.53% | 1.33% | motif file (matrix) |
